# Supplementary material for: Injectables’ key role in rifampicin-resistant tuberculosis shorter treatment regimen outcomes
Source: PLoS One. 2020 Aug 31;15(8):e0238016. doi: 10.1371/journal.pone.0238016 (PMC7458281; doi:10.1371/journal.pone.0238016)
Supplement: Attachment — Submitted filename: Response_to_reviewers_KM_2M.docx [file pone.0238016.s001.docx]

Dear Editor,

Many thanks for the review of "Injectables’ key role in rifampicin-resistant tuberculosis shorter treatment regimen outcomes". We have adapted the paper accordingly.

A revised manuscript showing modifications in track changes was uploaded, as well as a clean version and this rebuttal.

We shall of course be available to respond to any additional question you may have.

Kind regards,

Tom Decroo, on behalf of the co-authors

_____________________

PONE-D-20-18936
Injectables’ key role in rifampicin-resistant tuberculosis treatment outcomes
PLOS ONE

Dear Dr. Decroo,

Thank you for submitting your manuscript to PLOS ONE. Your manuscript was evaluated very positively by an independent referee and myself. Referee #1 raised a couple of minor concerns that should be addressed before we can proceed with formal acceptance. Therefore, we invite you to submit a revised version of the manuscript that addresses the points raised during the review process.

Please submit your revised manuscript by Sep 13 2020 11:59PM. If you will need more time than this to complete your revisions, please reply to this message or contact the journal office at [plosone@plos.org](mailto:plosone@plos.org). When you're ready to submit your revision, log on to <https://www.editorialmanager.com/pone/> and select the 'Submissions Needing Revision' folder to locate your manuscript file.

Please include the following items when submitting your revised manuscript:

- A rebuttal letter that responds to each point raised by the academic editor and reviewer(s). You should upload this letter as a separate file labeled 'Response to Reviewers'.
- A marked-up copy of your manuscript that highlights changes made to the original version. You should upload this as a separate file labeled 'Revised Manuscript with Track Changes'.
- An unmarked version of your revised paper without tracked changes. You should upload this as a separate file labeled 'Manuscript'.

If you would like to make changes to your financial disclosure, please include your updated statement in your cover letter. Guidelines for resubmitting your figure files are available below the reviewer comments at the end of this letter.

If applicable, we recommend that you deposit your laboratory protocols in [protocols.io](http://protocols.io) to enhance the reproducibility of your results. Protocols.io assigns your protocol its own identifier (DOI) so that it can be cited independently in the future. For instructions see: <http://journals.plos.org/plosone/s/submission-guidelines#loc-laboratory-protocols>

We look forward to receiving your revised manuscript.

Kind regards,

Olivier Neyrolles
Academic Editor
PLOS ONE

Journal requirements:

When submitting your revision, we need you to address these additional requirements.

1. Please ensure that your manuscript meets PLOS ONE's style requirements, including those for file naming. The PLOS ONE style templates can be found at

<https://journals.plos.org/plosone/s/file?id=wjVg/PLOSOne_formatting_sample_main_body.pdf> and

<https://journals.plos.org/plosone/s/file?id=ba62/PLOSOne_formatting_sample_title_authors_affiliations.pdf>

2. Thank you for stating in your ethics statement "The evaluation of the STR is covered by a study protocol approved by the National Research Ethics Committee of Bangladesh Medical Research Council and the Institutional Review Board of the Institute of Tropical Medicine Antwerp (1233/18)." Please clarify whether this specific study was fully approved by these ethics committees.

The approved protocols cover the objectives, data collection procedures, and analysis of the present study. We adapted the statement as follows: “The evaluation of the STR is fully covered by a study protocol approved by the National Research Ethics Committee of Bangladesh Medical Research Council and the Institutional Review Board of the Institute of Tropical Medicine Antwerp (1233/18).”

3. Please provide a summary table of patient demographics.

We added a summary table with age and gender, now “table 2” and added a sentence in the results text.

4.We note that you have indicated that data from this study are available upon request. PLOS only allows data to be available upon request if there are legal or ethical restrictions on sharing data publicly. For information on unacceptable data access restrictions, please see <http://journals.plos.org/plosone/s/data-availability#loc-unacceptable-data-access-restrictions>.

 In your revised cover letter, please address the following prompts:

 a) If there are ethical or legal restrictions on sharing a de-identified data set, please explain them in detail (e.g., data contain potentially identifying or sensitive patient information) and who has imposed them (e.g., an ethics committee). Please also provide contact information for a data access committee, ethics committee, or other institutional body to which data requests may be sent.

There are ethical and privacy concerns with sharing the dataset. Given the stigma associated with RR-TB disease and to maximally protect the patient’s privacy we prefer to not share the database in an unrestricted manner. Some specific medical data (combination of a rare outcome + age + gender +++ …) might enable re-identification, even though identifiers such as names and addresses were removed from the programme dataset prior to analysis.

We propose the following statement: “The data supporting the findings of this publication are retained at the Institute of Tropical Medicine, Antwerp and will not be made openly accessible due to ethical and privacy concerns. Data can however be made available after approval of a motivated and written request to the Institute of Tropical Medicine at [ITMresearchdataaccess@itg.be](mailto:ITMresearchdataaccess@itg.be)”

 b) If there are no restrictions, please upload the minimal anonymized data set necessary to replicate your study findings as either Supporting Information files or to a stable, public repository and provide us with the relevant URLs, DOIs, or accession numbers. Please see <http://www.bmj.com/content/340/bmj.c181.long> for guidelines on how to de-identify and prepare clinical data for publication. For a list of acceptable repositories, please see <http://journals.plos.org/plosone/s/data-availability#loc-recommended-repositories>.

 We will update your Data Availability statement on your behalf to reflect the information you provide.

 5.Thank you for stating the following in the Financial Disclosure section:

[The authors received no specific funding for this work.].

We added this statement under “Funding”.

 We note that one or more of the authors have an affiliation to the commercial funders of this research study : Independent Consultant

 1. Please provide an amended Funding Statement declaring this commercial affiliation, as well as a statement regarding the Role of Funders in your study. If the funding organization did not play a role in the study design, data collection and analysis, decision to publish, or preparation of the manuscript and only provided financial support in the form of authors' salaries and/or research materials, please review your statements relating to the author contributions, and ensure you have specifically and accurately indicated the role(s) that these authors had in your study. You can update author roles in the Author Contributions section of the online submission form.

 Please also include the following statement within your amended Funding Statement.

“The funder provided support in the form of salaries for authors [insert relevant initials], but did not have any additional role in the study design, data collection and analysis, decision to publish, or preparation of the manuscript. The specific roles of these authors are articulated in the ‘author contributions’ section.”

If your commercial affiliation did play a role in your study, please state and explain this role within your updated Funding Statement.

 2. Please also provide an updated Competing Interests Statement declaring this commercial affiliation along with any other relevant declarations relating to employment, consultancy, patents, products in development, or marketed products, etc.

 Within your Competing Interests Statement, please confirm that this commercial affiliation does not alter your adherence to all PLOS ONE policies on sharing data and materials by including the following statement: "This does not alter our adherence to  PLOS ONE policies on sharing data and materials.” (as detailed online in our guide for authors <http://journals.plos.org/plosone/s/competing-interests>). If this adherence statement is not accurate and  there are restrictions on sharing of data and/or materials, please state these. Please note that we cannot proceed with consideration of your article until this information has been declared.

Please include both an updated Funding Statement and Competing Interests Statement in your cover letter. We will change the online submission form on your behalf.

Please know it is PLOS ONE policy for corresponding authors to declare, on behalf of all authors, all potential competing interests for the purposes of transparency. PLOS defines a competing interest as anything that interferes with, or could reasonably be perceived as interfering with, the full and objective presentation, peer review, editorial decision-making, or publication of research or non-research articles submitted to one of the journals. Competing interests can be financial or non-financial, professional, or personal. Competing interests can arise in relationship to an organization or another person. Please follow this link to our website for more details on competing interests: <http://journals.plos.org/plosone/s/competing-interests>

AVD is an expert in the field of TB diagnosis and treatment. He worked for decades for Damien Foundation (DF) in their NGO medical aid projects, in one of which the study was conducted, and the Institute of Tropical Medicine Antwerp (ITM), where samples were send. Neither of those is of a commercial nature. Now he is retired. Given his expertise he still contributes to DF and ITM activities in general. Contribution to this specific study was not specified in the consultancy contract. He has no competing interests and no commercial affiliation. Therefore the statement “The authors received no specific funding for this work” applies to all authors.

Reviewers' comments:

Reviewer's Responses to Questions

**Comments to the Author**

1. Is the manuscript technically sound, and do the data support the conclusions?

The manuscript must describe a technically sound piece of scientific research with data that supports the conclusions. Experiments must have been conducted rigorously, with appropriate controls, replication, and sample sizes. The conclusions must be drawn appropriately based on the data presented.

Reviewer #1: Yes

2. Has the statistical analysis been performed appropriately and rigorously?

Reviewer #1: Yes

3. Have the authors made all data underlying the findings in their manuscript fully available?

The [PLOS Data policy](http://www.plosone.org/static/policies.action#sharing) requires authors to make all data underlying the findings described in their manuscript fully available without restriction, with rare exception (please refer to the Data Availability Statement in the manuscript PDF file). The data should be provided as part of the manuscript or its supporting information, or deposited to a public repository. For example, in addition to summary statistics, the data points behind means, medians and variance measures should be available. If there are restrictions on publicly sharing data—e.g. participant privacy or use of data from a third party—those must be specified.

Reviewer #1: Yes

4. Is the manuscript presented in an intelligible fashion and written in standard English?

PLOS ONE does not copyedit accepted manuscripts, so the language in submitted articles must be clear, correct, and unambiguous. Any typographical or grammatical errors should be corrected at revision, so please note any specific errors here.

Reviewer #1: Yes

5. Review Comments to the Author

Please use the space provided to explain your answers to the questions above. You may also include additional comments for the author, including concerns about dual publication, research ethics, or publication ethics. (Please upload your review as an attachment if it exceeds 20,000 characters)

Reviewer #1: The authors demonstrate in scientifically sound and convincing way the important role played by 2nd line injectables in preventing recurrence with acquired resistance to Gatifloxacine, the core drug in the hitherto most effective shorter treatment regimen (STR) of rifampicin resistant tuberculosis (RR-TB). Subsequently, they plead for the necessity to include in the evaluation of all-oral RR-TB regimens - promoted since 2019 by WHO alternatively to STRs including injectables - an assessment of regimen compositions on resistance acquisition. The study is an important contribution to the ongoing discussion about merit and virtue of the standardized ‘Bangladesh’ STR and its variants using injectables for programmatic management of RR-TB in L/MICs where treatment options after recurrence are limited and will be so in a foreseeable future.

Many thanks for your encouraging feedback

Minor suggestions

Title
Consider adding “…in RR-TB STR…”

The revised title reads as follows: “Injectables’ key role in rifampicin-resistant tuberculosis shorter treatment regimen outcomes”

Discussion
L289-90 Also understandable from the context, consider re-wording (…two days is not
appropriate for all TB drugs.” – “Two days of (what)…”.

The text was revised as follows: “Besides EBA estimates being prone to considerable variation, assessment at two days treatment is not appropriate for all TB drugs”

6. PLOS authors have the option to publish the peer review history of their article ([what does this mean?](https://journals.plos.org/plosone/s/editorial-and-peer-review-process#loc-peer-review-history)). If published, this will include your full peer review and any attached files.


If you choose “no”, your identity will remain anonymous but your review may still be made public.


**Do you want your identity to be public for this peer review?** For information about this choice, including consent withdrawal, please see our [Privacy Policy](https://www.plos.org/privacy-policy).

Reviewer #1: No

While revising your submission, please upload your figure files to the Preflight Analysis and Conversion Engine (PACE) digital diagnostic tool, <https://pacev2.apexcovantage.com/>. PACE helps ensure that figures meet PLOS requirements. To use PACE, you must first register as a user. Registration is free. Then, login and navigate to the UPLOAD tab, where you will find detailed instructions on how to use the tool. If you encounter any issues or have any questions when using PACE, please email PLOS at [figures@plos.org](mailto:figures@plos.org). Please note that Supporting Information files do not need this step.
